# MFSPSSMpred: identifying short disorder-to-order binding regions in disordered proteins based on contextual local evolutionary conservation

**DOI:** 10.1186/1471-2105-14-300

**Published:** 2013-10-04

**Authors:** Chun Fang, Tamotsu Noguchi, Daisuke Tominaga, Hayato Yamana

**Affiliations:** 1 Department of Computer Science and Engineering, Waseda University, Tokyo, Japan; 2 Computational Biology Research Center (CBRC), Tokyo, Japan; 3 Meiji Pharmaceutical University, Tokyo, Japan

**Keywords:** Molecular recognition features, Intrinsically disordered protein, Position-specific scoring matrix

## Abstract

**Background:**

Molecular recognition features (MoRFs) are short binding regions located in longer intrinsically disordered protein regions. Although these short regions lack a stable structure in the natural state, they readily undergo disorder-to-order transitions upon binding to their partner molecules. MoRFs play critical roles in the molecular interaction network of a cell, and are associated with many human genetic diseases. Therefore, identification of MoRFs is an important step in understanding functional aspects of these proteins and in finding applications in drug design.

**Results:**

Here, we propose a novel method for identifying MoRFs, named as MFSPSSMpred (Masked, Filtered and Smoothed Position-Specific Scoring Matrix-based Predictor). Firstly, a masking method is used to calculate the average local conservation scores of residues within a masking-window length in the position-specific scoring matrix (PSSM). Then, the scores below the average are filtered out. Finally, a smoothing method is used to incorporate the features of flanking regions for each residue to prepare the feature sets for prediction. Our method employs no predicted results from other classifiers as input, i.e., all features used in this method are extracted from the PSSM of sequence only. Experimental results show that, comparing with other methods tested on the same datasets, our method achieves the best performance: achieving 0.004~0.079 higher AUC than other methods when tested on TEST419, and achieving 0.045~0.212 higher AUC than other methods when tested on TEST2012. In addition, when tested on an independent membrane proteins-related dataset, MFSPSSMpred significantly outperformed the existing predictor MoRFpred.

**Conclusions:**

This study suggests that: 1) amino acid composition and physicochemical properties in the flanking regions of MoRFs are very different from those in the general non-MoRF regions; 2) MoRFs contain both highly conserved residues and highly variable residues and, on the whole, are highly locally conserved; and 3) combining contextual information with local conservation information of residues facilitates the prediction of MoRFs.

## Background

With the breaking of the conventional protein paradigm of “sequence-structure-function”, the functional importance of intrinsically disordered proteins (IDPs) has become increasingly apparent. Although IDPs have no well-defined tertiary structures in their natural state, they possess essential biological functions. IDPs play critical roles in a variety of physiological processes such as signal transduction, translation regulation, and protein modification. Specifically, in interaction-mediated signaling events, IDPs possess unique advantages [1]: (a) they lack a stable three-dimensional structure and their conformations can fluctuate over time to time; (b) they possess a combination of high specificity and low affinity; (c) they can recognize multiple partners through adoption of different conformations; (d) multiple distinct partners can bind to a common binding site of IDPs, where these partners may assume different folds. Many proteins from higher organism have been found to be entirely disordered or to contain partly disordered regions [2]. Due to the particular properties of IDPs, conventional structure determination methods are simply inapplicable to IDPs in isolation, because no stable structure exists. However, when bound to their molecular partners, many IDPs undergo a disorder-to-order transition [1-3]. This characteristic makes it possible to obtain the structures of IDPs by crystallizing them in complexes with their molecular binding partners.

Molecular recognition features (MoRFs) are short binding regions (5–25 residues) located in IDPs regions, which easily undergo disorder-to-order transitions upon binding to partner proteins [4]. According to their structures in the bound state, MoRFs can be divided into at least three sub-types: α-MoRFs, β-MoRFs, and ι-MoRFs, which form α-helices, β-strands, and structures without a regular pattern of backbone hydrogen bonds [2]. MoRFs are enrichment in highly connected hub proteins, and their complexity reinforces the functional importance of the disordered regions [3]. They act as molecular switches in molecular-interaction networks of the cell, and are assumed to be implicated in the causes of many diseases [1]. Thus, identification of MoRFs is a key step in understanding the functions of these proteins and in finding applications in drug design.

Experimental methods for identifying MoRFs are expensive and time consuming, which makes computational methods indispensable for guiding experimental analysis. So far, because of the limited number of experimentally validated MoRFs, only four custom-built tools for predicting MoRFs are available: α-MoRF-PredI [5] and α-MoRF-PredII [6] are neural network-based predictors aimed at predicting α-MoRFs; ANCHOR [7] concentrates on the prediction of MoRFs which bind to globular proteins; and MoRFpred [4] is a comprehensive predictor which combines the annotations generated by sequence alignments with the prediction results generated by a support vector machine (SVM). These predictors use a variety of predicted features as their input, including predicted disorder probabilities [4-7], predicted solvent accessibility [4], predicted secondary structure propensities [4,5], and predicted B-factors [4]. These predicted features not only easy to result in high-dimensional feature space, but also greatly increase the complexity of algorithms. Moreover, the performance of these predictors is also largely affected by other classifiers. Thus, a more simple and efficient method for identifying MoRFs is indispensable.

A number of studies have analyzed the attributes of MoRFs [1-9]. Norman et al. [3] found that several strong physicochemical preferences were shown in all MoRF types compared with in general disordered regions. Fuxreiter et al. [8] identified that amino acid composition and charge/hydropathy properties of MoRFs exhibited a mixture characteristic of folded and disordered structures. Chica et al. [9] found that the flanking regions of MoRFs were relevant to linear motif-mediated interactions at both the structural and sequence levels, and that the prediction of MoRFs can be facilitated by contextual information from the protein sequence.

Because the functional sites of proteins need to maintain a high degree of conservation to execute a given function, evolutionary information included in a position-specific scoring matrix (PSSM) has been considered as the most predictive feature for identifying the functional sites of ordered proteins. MoRFs are also found to be more conserved than their surrounding residues [9,10]. However, disordered proteins usually evolve more rapidly than ordered proteins; therefore, standard PSSMs which incorporate the conservation information of proteins are ineffective when used directly. Fortunately, relative local conservation has been proven to be a good feature for motif discovery, and has been used in a number of studies [10-12]. In addition, Shimizu et al. [13] found that ignoring some redundant features in standard PSSMs can improve the prediction of protein disordered regions significantly.

Taking the above information into account, we developed a novel sequence-based method for identifying MoRFs in IDPs. In this method, firstly, a masking method is used to calculate the average local conservative scores. Then, a filtering method is used to drop the scores below the average. Finally, a smoothing method is used to incorporate features of the flanking regions for each residue in the PSSM. The masking and filtering steps strengthen the highly conserved information and filter out poorly conserved information for each residue, thereby ensuring that only highly local conserved features are considered in the prediction. Moreover, the smoothing method can incorporate contextual information of neighboring residues for any given residue. All the related features are extracted only from protein sequences themselves. We used a support vector machine (SVM) to build the classifier.

## Methods

### Benchmark datasets

In the present study, one training dataset and three test datasets were adopted. We employed the same training dataset as the one that was used by Fatemeh et al. [4] in their study of MoRFpred. This dataset, called as TRAINING421, includes 421 MoRF-containing chains, that contained 5,396 positive samples (MoRF residues) and 240,588 negative samples (non-MoRF residues). All the positive samples and an equal number of randomly selected negative samples were used for training our prediction model. After that, three test datasets were used for testing the developed model.

First, the two test dataset used in the study of MoRFpred [4] were adopted.

#### TEST419

It included 419 MoRF-containing chains, which were deposited in PDB from April 2008, named as TEST419. They shared up to 30% sequence identity with the training dataset.

#### TEST2012

It included 45 MoRF-containing chains, which were deposited in PDB from January 1 to March 11, 2012 and in UniProtKB from February 22, 2012, named as TEST2012. They shared up to 30% sequence identity with the training dataset.

The training and the above two test datasets are available on the MoRFpred web server [http://biomine-ws.ece.ualberta.ca/MoRFpred/index.html] [4].

Because the TRAINING421 and TEST419 datasets were reported to contain a large number of immune response-related MoRFs (120 among the 840 MoRFs), we built another independent dataset to test whether the developed predictor was biased for some particular type of MoRFs.

#### TESTMem64

It included 64 non-redundant MoRF-containing membrane proteins (50 transmembrane proteins and 14 peripheral membrane proteins), which were extracted from the study of membrane proteins reported by Ioly et al. [14]. In their study, Ioly et al. collected 166 non-redundant MoRF-containing membrane protein sequences. We removed the sequences that either with sequence length >1000 residues, or with MoRF length >25 residues. Because sequences longer than 1000 residues cannot be processed by MoRFpred [4], in our study, we also aimed to predict short MoRFs (5–25 residues). After that, 64 sequences were retained, named as TESTMem64. Details of the TESTMem64 dataset are shown in Additional file 1: Table S1.

### Feature analysis of MoRFs, their flanking regions, and general non-MoRF regions

#### Composition analysis

The composition of the 421 protein sequences related to TRAINING421dataset was analyzed. The sequences were characterized into three regions: MoRF region, regions flanking the MoRFs, and general non-MoRF regions.

**Figure 1:**
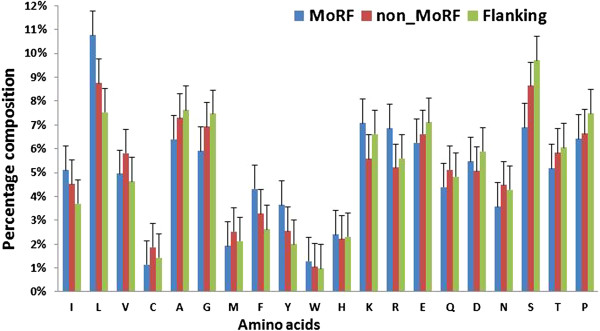
Distribution of the amino acids in three regions: MoRF regions (blue), 5-residue long flanking regions (green), and non-MoRF regions (red).

When the length of the flanking region was 5-residue long, amino acid composition of the three regions is shown in Figure 1. The composition of the MoRFs and their flanking regions deviated significantly from that of the general non- MoRF regions. The MoRFs were enriched in hydrophobic residues Ile, Leu, Phe, Tyr and Lys, as well as in charged residues Arg, and Asp, while depleted in Ala and Gly. The flanking regions were enriched in variable (disorder-promoting) amino acids, such as Ala, Gly, Glu, Ser and Thr, and depleted in hydrophobic and rigid (order-promoting) residues. Both of MoRFs and the flanking regions exhibit a large excess of a small, polar residue--Serine. The general non-MoRF regions are overrepresented with Val, Cys, Ala, Gly, Asn, and Ser, most of which were small and tiny amino acids.

**Figure 2:**
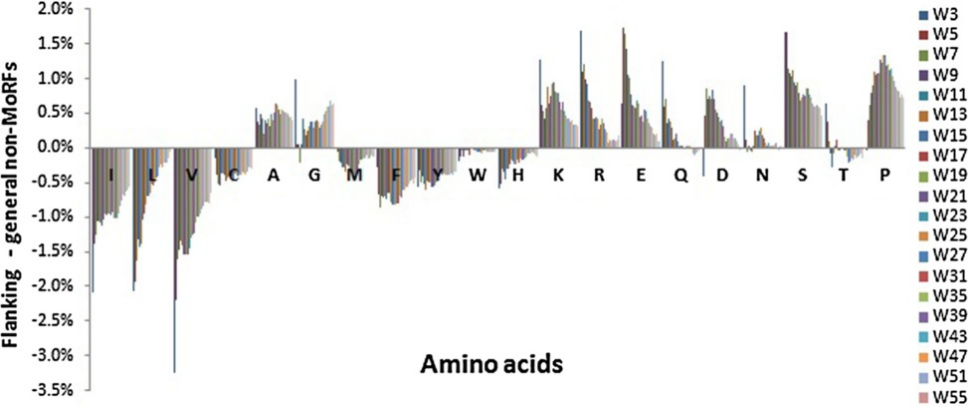
**Differences in amino acid composition between MoRF flanking and non-MoRF regions.** W indicates the length of the flanking regions; for example, W5 indicates a 5-residue long MoRF flanking region.

We further analyzed the composition of flanking regions with different lengths. While the composition distribution of general non-MoRF regions was essentially unchanged over different lengths (see the Additional file 2: Figure S1), that of the flanking regions changed significantly (see the Additional file 3: Figure S2). The composition differences between the flanking regions and the general non-MoRF regions (% amino acids in flanking regions - % amino acids in general non-MoRF regions) are shown in Figure 2. We also calculated the Pearson product–moment correlation coefficients between the flanking length and the composition difference for each amino acid (Additional file 4: Table S2). The correlation coefficients related to Ile, Leu, Val, His, Arg, Glu and flanking regions Ser have absolute value > 0.75 at the *p*-value <0.001. It illustrates that, composition of these amino acids in the flanking regions and in the general non-MoRF regions are significantly different.

#### Physicochemical properties

**Figure 3:**
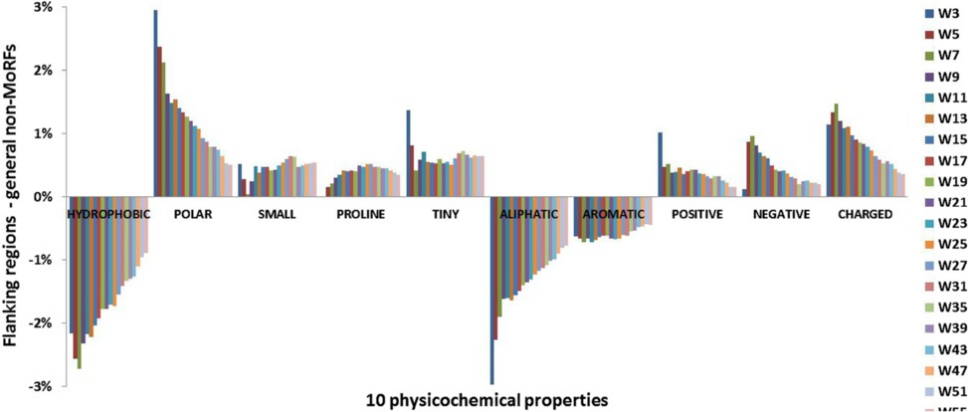
**Differences in physicochemical properties between MoRF flanking and non-MoRF regions.** W indicates the length of the flanking regions; for example, W5 indicates a 5-residue long MoRF flanking region.

We next analyzed differences between flanking and general non-MoRF regions with respect to ten physicochemical properties, namely: hydrophobic, polar, small, proline, tiny, aliphatic, aromatic, positive, negative and charged. The difference of physicochemical propensity between the flanking regions and general non-MoRF regions (property percentage in flanking regions - property percentage in general non-MoRF regions) is shown in Figure 3. The corresponding Pearson correlation coefficients between the flanking length and each physicochemical properties difference are shown in Additional file 5: Table S3. Correlation coefficients related to hydrophobic, polar, aliphatic, aromatic, positive, negative and charged have absolute value > 0.75 at the *p*-value <0.001. This phenomenon illustrates that, physicochemical properties of flanking regions are very different from those of general non-MoRF regions, namely that, the arrangements and properties of neighboring residues significantly impact the plasticity of MoRFs [8].

The analyses of the composition and physicochemical properties for the three regions illustrated that flanking regions are highly relevant to the MoRFs. Therefore, we assumed that the MoRFs in protein sequences are highly contextual. Because MoRFs are found to be more conserved than surrounding residues [9,10], we considered incorporating contextual information of residues with local evolutionary conservation to improve the prediction of MoRFs.

### Evolutionary information from PSSM

Evolutionary information was obtained from PSSMs, which were generated by PSI-BLAST [15], searching against the NCBI non-redundant (nr) database [16] by three-time iteration with an e-value of 0.001. Evolutionary information for each amino acid was encapsulated in a vector of 20 dimensions, where the size of PSSM matrix of a protein with N residues is 20 × N. 20 dimensions were considered as a standard amino acid size, and N is the length of the protein sequence.

### Masking and filtering the PSSM

Masked PSSM was used to describe the relative local evolutionary information of each residue in a protein. It was converted from a standard PSSM according to Formula (1).

(1) $$\mathrm{Masking}\_C_{i}=C_{i}-\frac{1}{2n+1}\sum_{i-n}^{i+n}C_{j},\left(i=1,\ldots ,N,\mathrm{where}\;N\;\mathrm{is}\;\mathrm{the}\;\mathrm{sequence}\;\mathrm{length}\right),$$

where *Masking*_*C*_*i*_ represents the relative local conservation score of residue *i*, *Ci* is the standard conservation score in the PSSM, and *2n+1* is the masking-window size.

In order to extract highly locally conserved information and filter out poorly locally conserved information from a sequence, the masked PSSM was further converted to a filtered PSSM according to Formula (2). All the scores below the mean score within a masking-window length were set to 0.

(2) $$\begin{array}{ll} \mathrm{Filtering}\_C_{i}= & \left\{\begin{array}{l} \mathrm{Masking}\_C_{i},\left(\mathrm{Masking}\_C_{i}>0\right)\\ 0,\left(\mathrm{Masking}\_C_{i}\leq 0\right) \end{array}\right),\\ & \left(i=1,\ldots ,N,\mathrm{where}\;N\;\mathrm{is}\;\mathrm{the}\;\mathrm{sequence}\;\mathrm{length}\right) \end{array}$$

**Figure 4:**
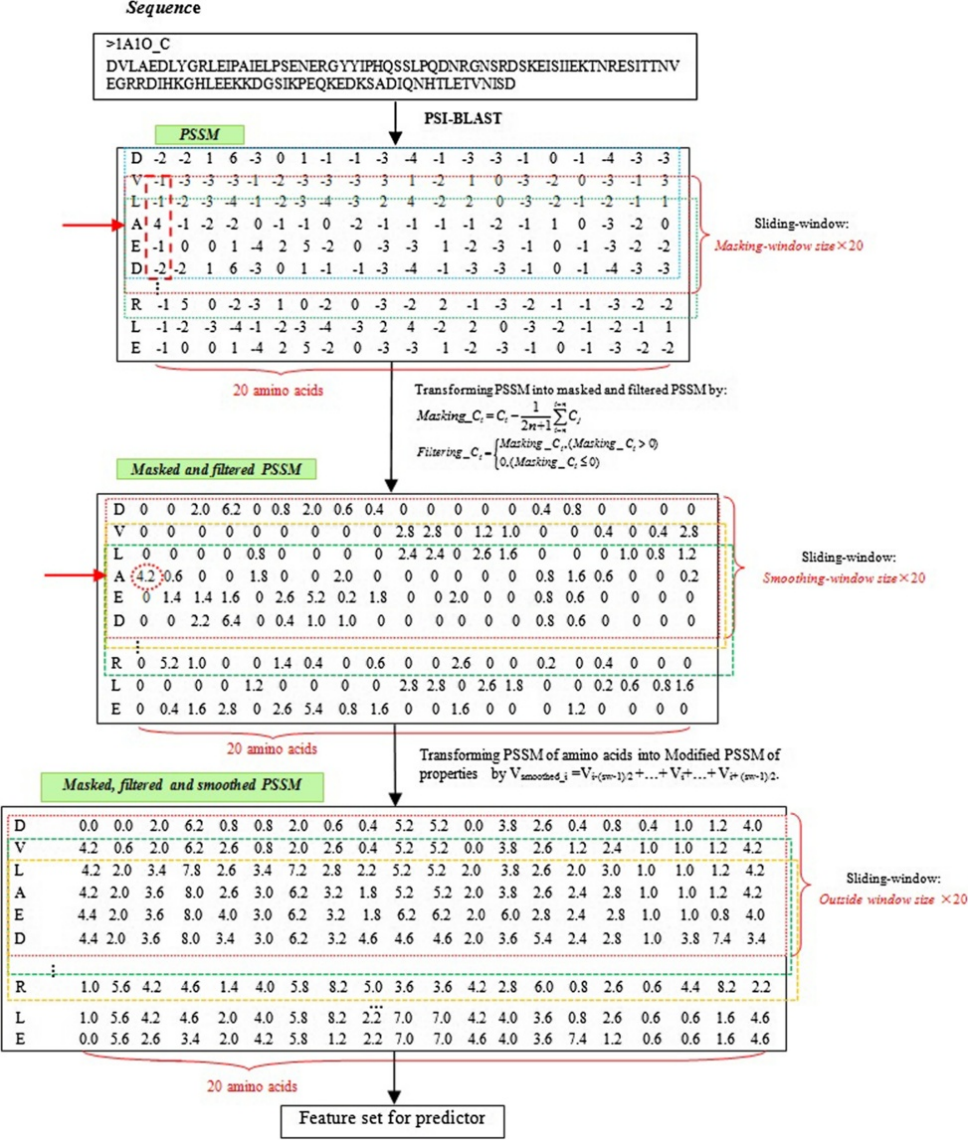
Procedure of preparing feature sets for the predictor.

An example of a masked and filtered PSSM profile is shown in Figure 4. For amino acid 'A', the first column of the vector was masked by 4 - [(-1) + (-1) + 4 + (-1) + (-2)]/5 = 4.2, because 4.2 > 0, it was retained, otherwise, it would be set to 0. After modification, only the scores that represented strong local conservation were retained.

### Smoothing the modified PSSM

Every value in a standard PSSM is calculated based on the assumption that the position of each value in the matrix is independent of the others. However, residues in MoRF regions appear continuously and the plasticity of MoRFs is also largely affected by neighboring residues [8]. Accordingly, in order to incorporate the dependency on surrounding neighbors of a central residue, we adopted a smoothing method [17] that was inspired by the smoothing skill used in image processing [18]. Firstly, to address the N-terminal and C-terminal of a protein sequence, *m* (*m* is an odd number) ZERO vectors were appended to the head and tail of a standard PSSM profile, where *2m+1* was the size of a smoothing sliding-window. The smoothing sliding-window was then used to incorporate the evolutionary information from the upstream and downstream residues. Each row vector of an amino acid residue *C*_*i*_ was smoothed according to Formula (3). Finally, each value in the masked and smoothed PSSM matrix was scaled to a range of [-1, 1] according to a certain ratio.

(3) $$\begin{array}{l} \mathrm{Smoothing}\_C_{i}=\sum_{i-m}^{i+m}\mathrm{Filtering}\_C_{j},\\ \left(i=1,\ldots ,N,N\;\mathrm{is}\;\mathrm{the}\;\mathrm{sequence}\;\mathrm{length},2m+1\;\mathrm{is}\;\mathrm{smoothing}-\mathrm{window}\;\mathrm{size}\right) \end{array}$$

### Prediction model

**Figure 5:**
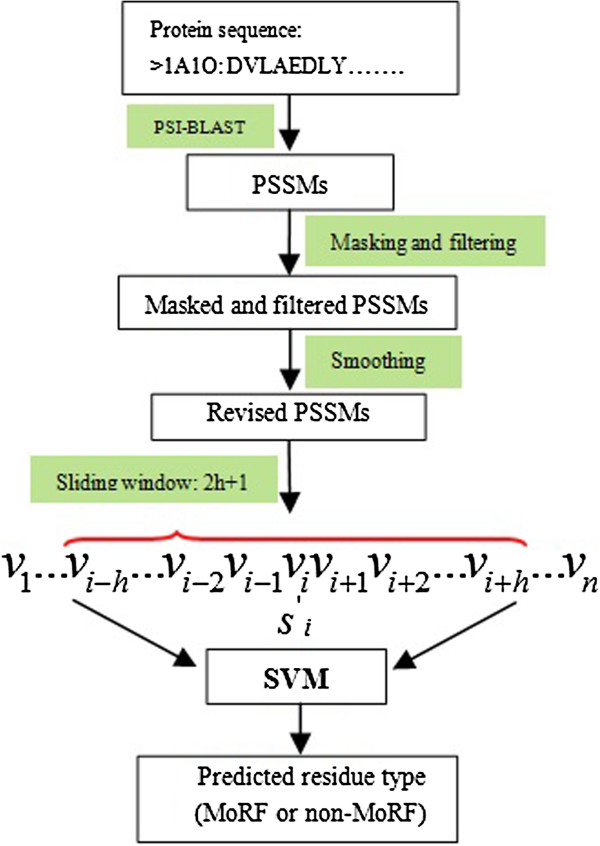
**Prediction model design.***2h+*1 is the length of the outside sliding-window, *n* is the sequence length, and *v*_*i*_ represents the corresponding amino acids *i* in the feature vector.

We adopt a modified PSSM, which incorporates the information of amino acid position, relative evolutionary information, and dependency on neighboring residues, to design our prediction model. Only highly conserved position scores calculated from PSSMs were considered in our prediction. Firstly, a masking method was used to calculate the average score of the local conservation within a masking-window length. Next, scores lower than the averages were filtered out. This procedure could strengthen the highly locally conserved information and filter out poorly locally conserved information of residues. Finally, a smoothing method was adopted to incorporate feature information from the neighbors flanking each residue. The prediction model is shown in Figure 5, and the detailed procedure for preparing the input feature set is shown in Figure 4.

### SVM

Prediction of MoRFs can be addressed as a two-classification problem, namely, determining whether a given residue belongs to a MoRFs or not. Our prediction model was trained using the LIBSVM software package [19,20]. Here, the Radial Basis Function (RBF kernel) was selected as the kernel function. The capacity parameter *c* and kernel width parameter *g* were then optimized using a grid search approach [19,20].

### Evaluation criteria

We adopted the evaluation criteria used in the 10th Critical Assessment of Techniques for Protein Structure Prediction (CASP10) experiment [21]. The area under ROC curve (AUC) and the accuracy (ACC) were adopted to evaluate the performance of the classifiers. Receiver operating characteristic (ROC) plots with AUC values were generated using the R statistical package [22]. The sensitivity, specificity, true positive rate (TPR), false negative rate (FPR), success rate, and ACC were defined as follows:

(4) $$\mathrm{Specifity}=\frac{\mathrm{TN}}{\mathrm{TN}+\mathrm{FP}}$$

(5) $$\mathrm{TPR}=\mathrm{Sensitivity}=\frac{\mathrm{TP}}{\mathrm{TP}+\mathrm{FN}}$$

(6) $$\mathrm{FPR}=1-\mathrm{Specificity}=\frac{\mathrm{FP}}{\mathrm{TN}+\mathrm{FP}}$$

(7) $$\mathrm{Accuracy}=\frac{\mathrm{TP}+\mathrm{TN}}{\mathrm{TP}+\mathrm{TN}+\mathrm{FP}+\mathrm{FN}}$$

(8) $$\mathrm{ACC}=\frac{1}{2}\left(\mathrm{Sensitivity}+\mathrm{Specificity}\right)$$

Where TP, TN, FP and FN represents true positive, true negative, false positive and false negative respectively.

## Results and discussion

### Optimizing window size

**Figure 6:**
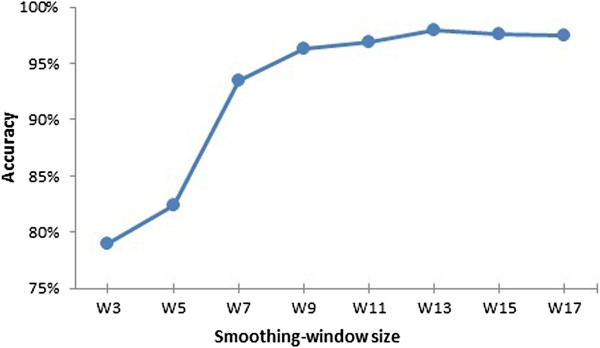
**Cross-validation accuracy of MFSPSSMpred with different smoothing-window sizes.** W indicates the length of windows, i.e., W3 means 3-residue long.

To develop the MFSPSSMpred model, three window sizes were necessary: (i) the outside sliding-window size which ultimately determined the dimensions of feature vectors; (ii) the masking-window size which was used to calculate the average conservation scores in a local region; and (iii) the inside smoothing-window size which was used to strengthen locally conserved features. To make a fair comparison among the different predictors, we chose the same outside-sliding window size of '25’ with the research MoRFpred [4]. The masking-window size was also assigned to '25’, since it has the similar meaning with the outside sliding-window size, both of which indicated the flanking length that would be considered to affect a central residue. Here, because the average scores calculated from different lengths greater than 10 residues were highly similar, we found that variations in masking-window size had limited influence on the results (Results of MFSPSSMpred with different masking-window sizes are shown in Additional file 6: Figure S3). Next, MFSPSSMpred models with different smoothing-window sizes were tested by 5-cross-validation using the grid search approach [19]. The cross-validation accuracies according to different smoothing-window sizes are shown in Figure 6. The predictor performance stabilized with the smoothing-window sizes greater than 9. Therefore, we chose the relatively best size 13 as the inside smoothing-window size for our model.

### Effectiveness of the feature extracting methods

#### Effectiveness at the individual protein level

To confirm the effectiveness of our feature extracting method in distinguishing MoRF residues from non_MoRF residues and to determine how this might benefit the predication, we selected the yeast elongin C complex with a von Hippel-Lindau peptide [PDB: 1HV2] as an example. Yeast elongin C is a signaling protein, which is highly intrinsically disordered and has a MoRF located at the region of residues 100–212 of its B chain.

**Figure 7:**
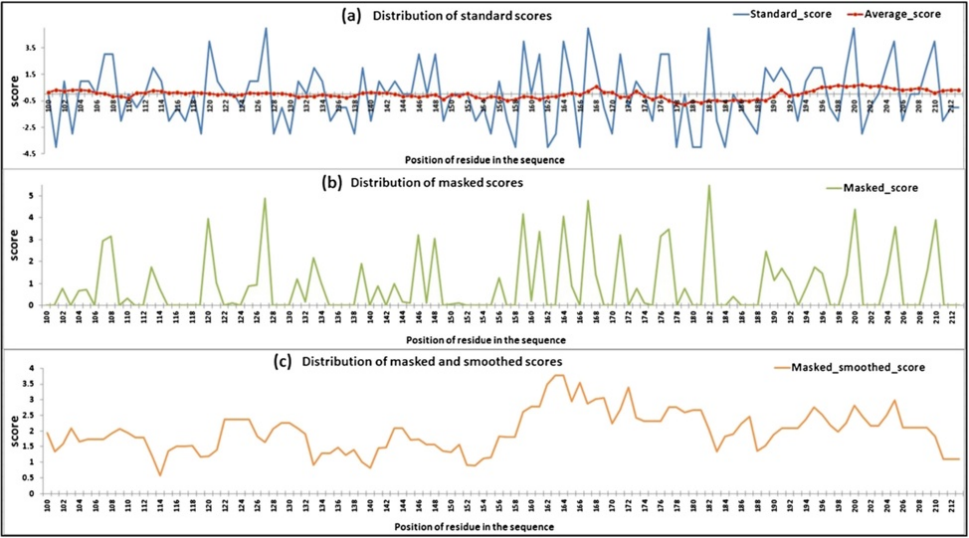
**Distribution of the conservation scores for protein 1HV2 (PDB ID) related to the standard PSSM (a), masked PSSM (b), masked and smoothed PSSM (c).** The red-dotted rectangles mark the position of MoRF (only residues 100~212 in the **B** chain are displayed).

We extracted a column of position-specific scores randomly from the standard PSSM (there are a total of 20 columns, corresponding to the 20 standard amino acids). Then, the distribution of the scores in the standard PSSM, masked PSSM, and smoothed PSSM, was counted (Figure 7), in the standard PSSM, the distribution of scores between the MoRF and non_MoRF showed no distinct difference. The MoRFs regions included both highly conserved residues and highly variable residues, which is consistent with the property of MoRFs. i.e., to maintain a certain function, MoRFs must be highly conserved. On the other hand, MoRFs must be highly variable because of their intrinsically disordered nature. In the masked PSSM, scores below the average have been filtered out, thereby discarding the noise data (low conservative scores), that are undesirable for prediction. In the smoothed PSSM, scores of residues that are surrounded by highly conserved residues have been enhanced after smoothing, and scores of residues that are surrounded by poorly conserved residues have been weakened. Since MoRFs are usually composed of intensively conserved residues, they are easy to appear as highly conserved peaks compared with the non_MoRF regions. The results demonstrate that, after masking and smoothing the PSSM, the position-specific scores in MoRF regions become significantly higher than those in the non_MoRF regions. Here, we have shown the results for just one column of scores in the PSSM, similar results were obtained for the scores in the other 19 columns of the PSSM (data not shown).

### Effectiveness at the whole dataset level

We also calculated the distributions of local conservation scores in each of the three PSSMs for all residues in the 840 chains (TRAINING421 and TEST419). We assumed that the summation of scores in each row of the corresponding PSSMs represented the local conservation of the related residues. The local conservation scores in each PSSM were calculated according to Formulas (9), (10) and (11).

(9) $$\mathrm{Sum}\_\mathrm{Original}\_C_{i}=\sum_{j=1}^{20}\mathrm{Original}\_C_{j},\left(i=1,\ldots ,N,N\;\mathrm{is}\;\mathrm{the}\;\mathrm{sequence}\;\mathrm{length}\right)$$

(10) $$\mathrm{Sum}\_\mathrm{Masked}\_C_{i}=\sum_{j=1}^{20}\mathrm{Masked}\_C_{j},\;\left(i=1,\ldots ,N,\;N\;\mathrm{is}\;\mathrm{the}\;\mathrm{sequence}\;\mathrm{length}\right)$$

(11) $$\mathrm{Sum}\_\mathrm{Smoothed}\_C_{i}=\sum_{j=1}^{20}\mathrm{Smoothed}\_C_{j},\left(i=1,\ldots ,N,N\;\mathrm{is}\;\mathrm{the}\;\mathrm{sequence}\;\mathrm{length}\right)$$

where '20’ is the number of standard amino acid residues, and also the number of columns in the PSSMs (PSSM is a 20 × *N* matrix), and *N* is the sequence length.

**Figure 8:**
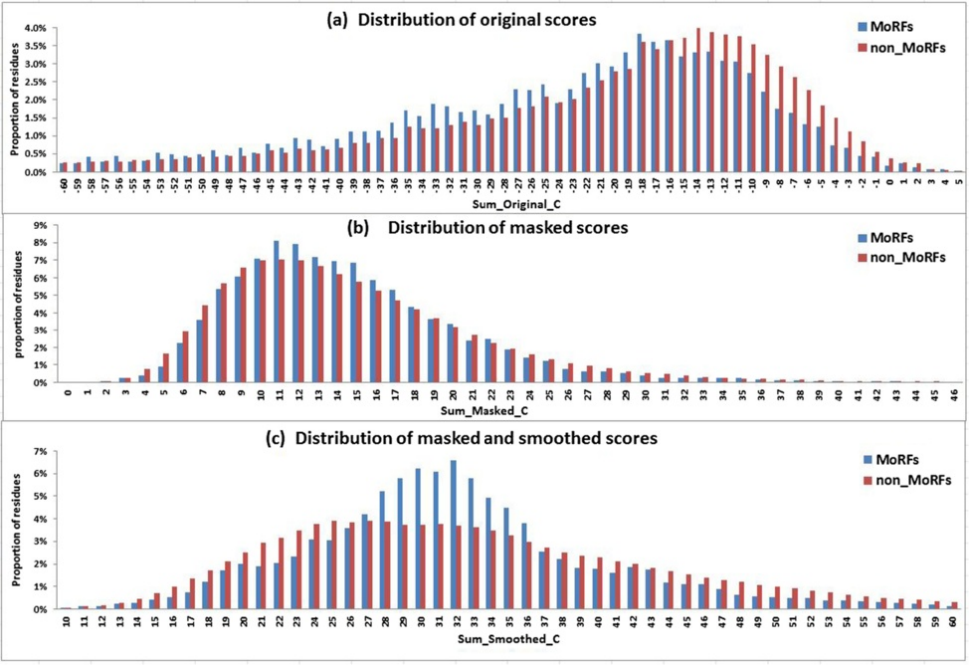
**Distribution of the summation scores of residues in MoRF and non-MoRF regions related to the standard PSSM (a), masked PSSM (b), masked and smoothed PSSM(c).** Here the non-MoRF regions include the residues in the flanking regions.

We compared the distribution of the amino acid residues in the MoRF and non_MoRF regions in each of the three PSSMs. In the standard PSSM, the MoRF residues are at a disadvantage in conservation compared with the non_MoRF residues (Figure 8a), because MoRFs contain highly variable residues. After masking and filtering, the differences between the residues in the MoRF and non_MoRF regions became less obvious, and some MoRF residues exhibited more strongly conserved than the non_MoRF residues (Figure 8b), showing that most of the filtered scores belonged to the non_MoRF residues. Finally, after smoothing, the residues in the two regions had distinctly different distributions; the MoRF residues were much more strongly conserved than most of non_MoRF residues (Figure 8c). The findings obtained using the whole of the TRAINING421 and TEST419 datasets show that our feature extracting method can effectively distinguish MoRF residues from non_MoRF residues as illustrated in Figure 8.

### Performance tested on TEST419, TEST2012 and comparison with other feature-based methods

**Figure 9:**
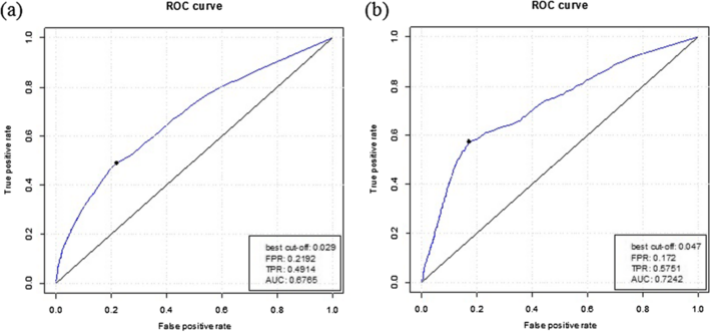
ROC plots of MFSPSSMpred tested on TEST419 (a) and TEST2012 (b).

First, TRAINING421 was used to train MSPSSMpred, which was then tested on the TEST419 and TEST2012 datasets. ROC plots of the results are shown in Figure 9(a ~b) respectively. For TEST419, the AUC was 0.677 and, for TEST2012, the AUC was 0.724.

Originally, the direct outputs of PSSMs from PSI-BLAST have provided conversation information by default and have been widely used to predict various protein functional sites. However, there is a room for improvement, because standard PSSMs contain redundant features.

**Figure 10:**
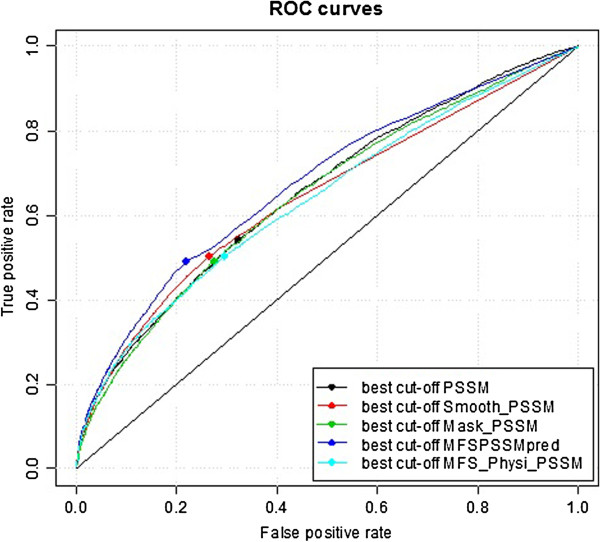
ROC plots for MFSPSSMpred and four other PSSM-based methods.

Here, we have compared our method with four other PSSM_based methods: 1) the 'PSSM’ method, which uses the standard PSSM (the direct output of PSSMs) for prediction; 2) the 'Smooth_PSSM’ method, which uses smoothed PSSMs without masking and filtering; 3) the 'Mask_PSSM’ method, which uses masked and filtered PSSMs without smoothing; and 4) the 'MFS_Physi_PSSM’ method, which is similar to our MFSPSSMpred method but incorporates 10 physicochemical properties of residues as input. The performances of MFSPSSMpred and the other four methods based on TRAINING421 and TEST419 are shown in Table 1, and ROC plots for all the methods are shown in Figure 10. The results demonstrate that MFSPSSMpred achieves the best performance.

**Table 1:** Performance comparison with four other PSSM-based methods

**Test dataset**	**Methods**	**ACC**	**TPR**	**FPR**	**AUC**
TEST419	PSSM	0.610	0.542	0.322	0.655
	Smooth_PSSM	0.620	0.503	0.264	0.644
	Mask_PSSM	0.609	0.492	0.273	0.648
	**MFSPSSMpred**	**0.636**	**0.491**	**0.219**	**0.677**
	MFS_Physi_PSSM	0.604	0.503	0.294	0.639

All methods used the same outside sliding-window size of 25. Smooth_PSSM and Mask_PSSM adopted the same smoothing or masking-window size with MFSPSSMpred.

### Performance comparison with existing predictors

**Figure 11:**
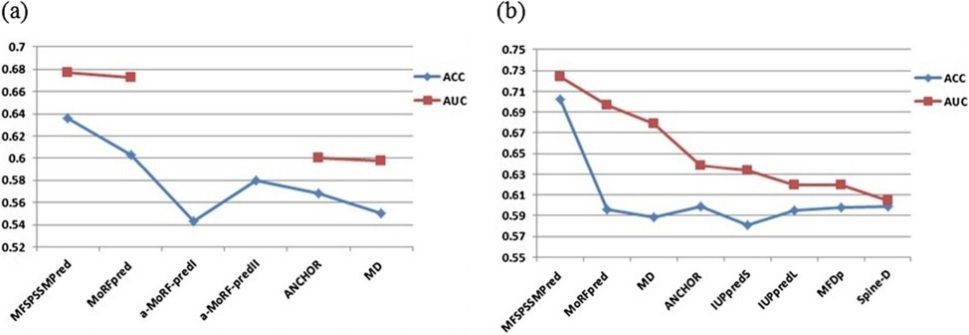
**ROC and ACC for all the predictors tested on TEST419 (a) and TEST2012 (b).** The AUC cannot be computed for α-MoRF-predI and α-MoRF-predII because they generate only binary predictions.

Some existing tools that are publicly available for MoRFs prediction have been tested on the TEST419 and TEST2012 datasets [4]. Here, we list them out for a comparison. Results are shown in Table 2, and the results of other classifiers are quoted from the study of Fatemeh et al. [4]. Details of ROC and ACC for all the predictors are shown in Figure 11. The results demonstrate that, MFSPSSMpred outperformed the other predictors with respect to ACC and AUC on both the TEST419 and TEST2012 datasets.

**Table 2:** Performance comparisons tested on the TEST419 and TEST2012 datasets

**Test dataset**	**predictor**	**ACC**	**TPR**	**FPR**	**AUC**
TEST419	MFSPSSMPred	**0.636**	0.491	0.219	**0.677**
	MoRFpred [4]	**0.603**	0.254	0.049	**0.673**
	α-MoRF-predI [5]	**0.543**	0.123	0.037	NA*
	α-MoRF-predII [6]	**0.580**	0.258	0.098	NA*
	ANCHOR [7]	**0.568**	0.389	0.253	**0.600**
	MD [23]	**0.550**	0.485	0.386	**0.598**
TEST2012	MFSPSSMPred	**0.702**	0.575	0.172	**0.724**
	MoRFpred [4]	**0.596**	0.236	0.045	**0.697**
	MD [23]	**0.589**	0.613	0.436	**0.679**
	ANCHOR [7]	**0.599**	0.433	0.236	**0.638**
	IUPpredS [24]	**0.581**	0.449	0.287	**0.634**
	IUPpredL [24]	**0.595**	0.572	0.382	**0.62**
	MFDp [25]	**0.598**	0.752	0.556	**0.62**
	Spine-D [26]	**0.599**	0.72	0.522	**0.605**
	DISOPRED2 [27]	**0.544**	0.543	0.455	**0.548**
	DISOclust [28]	**0.530**	0.653	0.593	**0.512**

* Because the α-MoRF-predI and α-MoRF-predII generate only binary predictions, their AUC cannot be computed.

### Performance on unbalanced training samples

**Figure 12:**
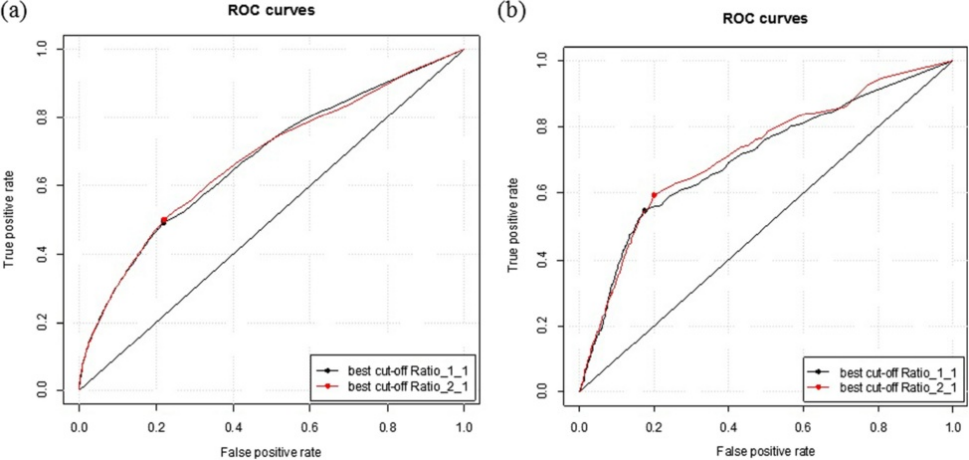
**Performance of MFSPSSMpred on different ratios of training samples, tested on TEST419 (a) and TEST2012 (b).** Red plots represent the result based on a training dataset with 1:2 ratio of MoRFs to non_MoRFs; the black plots represent the result based on a training dataset with 1:1 ratio of MoRFs to non_MoRFs.

The TRAINING421 dataset contains 5,601positive samples and 262,732 negative samples, and the ratio between them is 1:46.9. In order to analyze whether this imbalance biased the prediction method, we developed another training model with a 1:2 ratio between the MoRF and non-MoRF residues, that is, 5,601 MoRFs with 112,02 non-MoRF residues, and tested it on the TEST419 and TEST2012 datasets (Figure 12(a ~b)). Our results demonstrate that there was no significant difference in the performance between the 2:1 and 1:1 ratios.

### Performance tested on TESTMem64 and comparison with MoRFpred

**Figure 13:**
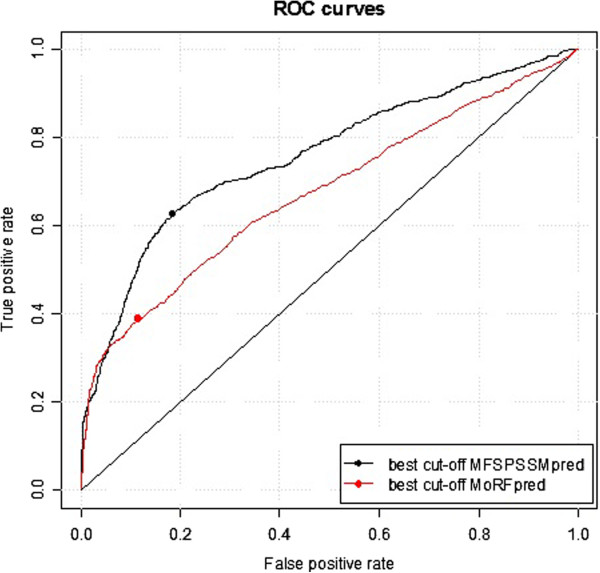
ROC plots of MFSPSSMpred (black) and MoRFpred (red) tested on TESTMem64.

The TRAINING421 and TEST419 datasets contain a large proportion (120 among the 840 MoRFs) of immune response-related MoRFs [4]. In order to test whether MFSPSSMpred was biased for some particular type of MoRFs, we built an independent test dataset --TESTMem64, which was extracted from another independent study of membrane proteins by Ioly et al. [14]. MoRFpred [4] was also tested on TESTMem64 for a comparison. Results are shown in Figure 13 and a detailed comparison of ACC, TPR, FPR and AUC is shown in Table 3. MFSPSSMpred performed much better than MoRFpred, achieving significantly higher ACC and AUC than MoRFpred.

**Table 3:** Performance comparison between MFSPSSMPred and MoRFpred tested on TESTMem64

**Test dataset**	**Method**	**ACC**	**TPR**	**FPR**	**AUC**
TESTMemMoRFs	MFSPSSMpred	**0.722**	0.627	0.185	**0.758**
	**MoRFpred**	**0.638**	0.389	0.114	**0.674**

We speculate that the reasons for the better performance of MFSPSSMpred include: (1) the MoRFpred method incorporated many predicted results, such as predicted disorder probabilities, predicted B-factor and predicted relative solvent accessibility derived from other predictors, as input for the prediction. These predicted features themselves are largely affected by the other classifiers those were used. Moreover, incorporating many predicted features can easy to result in a high-dimensional feature space; (2) MoRFpred [4] merges the result generated by an SVM and the result generated by sequence alignment with the MoRFs database into their final prediction result. Since there are so many immune response-related MoRFs in their database, MoRFpred is inevitably biased towards this type of MoRFs; (3) MFSPSSMpred used only the PSSM as input for prediction. It caught the point that, MoRF regions in a sequence are mingled with highly conserved residues and highly variable residues. Therefore, our approach is independent of the type or binding partners of MoRFs, and can be applied to the prediction of all MoRFs.

## Conclusions

In this study, we propose a novel method which adopts a modified PSSM encoding scheme for MoRFs prediction. Our method employs no predicted results as input, and all input features are extracted only from the PSSMs of sequences. By means of masking, filtering and smoothing, the modified PSSMs combine predictive features, which can effectively distinguish MoRF from non_MoRF residues. When comparing with other existing methods on the same datasets, MFSPSSMpred outperformed them all, achieving 3.3%~9.3% higher ACC and 0.004~0.079 higher AUC than other methods when tested on TEST419, and 10.3%~17.2% higher ACC and 0.045~0.212 higher AUC than other methods when tested on TEST2012. Moreover, despite the training dataset being biased toward immune response-related proteins, when MFSPSSMpred was tested on an independent membrane proteins-related dataset---TESTMem64, it showed good adaptability and significantly outperformed the existing MoRFpred predictor [4].

In summary, this study shows that combining contextual information with local conservation information of residues is predictive for identifying MoRFs. In addition, our study revealed some hallmarks of MoRFs; namely, MoRFs are mingled with highly conserved residues and highly variable residues, and MoRFs, on the whole, are highly locally conserved and are flanked by less conserved residues. A free Web server has been developed, which allows users to identify MoRFs in a given sequence using the model trained on our dataset. It is available from URL http://webapp.yama.info.waseda.ac.jp/fang/MoRFs.php.

## Abbreviations

MoRFs: Molecular recognition features; PSSM: Position-specific scoring matrix; SVM: Support vector machine; IDPs: Intrinsically disordered proteins; ROC: Receiver operating characteristic; AUC: Area under the corresponding ROC curve; TPR: True positive rate; FPR: False negative rate.

## Competing interests

The authors declare that they have no competing interests.

## Authors’ contributions

C.F carried out the implementation and drafted the manuscript, T.N and H.Y read and revised the final manuscript, all authors read and approved the final manuscript.

## Supplementary Material

Additional file 1: Table S1 Information about the TESTMem64 dataset. Information of binding partners is included. Click here for file

Additional file 2: Figure S1 Composition distribution of general non-MoRF regions with different flanking-window lengths (excluding flanking regions, W indicates the flanking window length). Click here for file

Additional file 3: Figure S2 Composition distribution of the flanking regions. W indicates the flanking window length. Click here for file

Additional file 4: Table S2 Correlation coefficients between flanking length and composition difference (Flanking regions - general non-MoRF regions) for each amino acid. Click here for file

Additional file 5: Table S3 Correlation coefficients between flanking length and physicochemical properties difference (Flanking regions - general non-MoRF regions) for each property. Click here for file

Additional file 6: Figure S3 ROC plots of MFSPSSMpred tested with different masking-window sizes (from 3 to 19). Click here for file
