# Supplementary material for: MFSPSSMpred: identifying short disorder-to-order binding regions in disordered proteins based on contextual local evolutionary conservation
Source: BMC Bioinformatics. 2013 Oct 4;14:300. doi: 10.1186/1471-2105-14-300 (PMC3853019; doi:10.1186/1471-2105-14-300)
Supplement: Additional file 1: Table S1 — Information about the TESTMem64 dataset. Information of binding partners is included. [file 1471-2105-14-300-S1.pdf]

## The TESTMem64 dataset

| | Uniprot Accession (MoRF-containing protein) | MoRF's length | MoRF's start (in Uniprot) | MoRF's end (in Uniprot) | MoRF's Secondary structure type | Uniprot Accession (Partner) |
|---|---|---|---|---|---|---|
| 1 | P01892 | 15 | 127 | 141 | i-MoRF | P01903<br>P04229 |
| 2 | P05504 | 13 | 29 | 41 | i-MoRF | P16391 |
| 3 | Q14118 | 15 | 881 | 895 | i-MoRF | P11532 |
| 4 | P25942 | 20 | 247 | 266 | i-MoRF | Q13114 |
| 5 | P16410 | 11 | 197 | 207 | i-MoRF | Q96CW1 |
| 6 | P16109 | 18 | 813 | 830 | i-MoRF | P84092 |
| 7 | P07204 | 19 | 426 | 444 | i-MoRF | P00734 |
| 8 | P35330 | 16 | 253 | 268 | i-MoRF | P26043 |
| 9 | P20645 | 13 | 265 | 277 | i-MoRF | Q9NZ52 |
| 10 | Q13291 | 11 | 276 | 286 | i-MoRF | O60880 |
| 11 | P05106 | 12 | 765 | 776 | i-MoRF | P54939 |
| 12 | Q01590 | 10 | 201 | 210 | i-MoRF | P40482 |
| 13 | P0A921 | 13 | 33 | 45 | α-MoRF | P0A921 |
| 14 | P36941 | 24 | 385 | 408 | i-MoRF | Q13114 |
| 15 | P56817 | 12 | 490 | 501 | i-MoRF | Q9UJY5 |
| 16 | P11362 | 22 | 409 | 430 | i-MoRF | Q8WU20 |
| 17 | O15162 | 10 | 257 | 266 | i-MoRF | P52293 |
| 18 | P31431 | 17 | 182 | 198 | i-MoRF | O00560 |
| 19 | P51577 | 10 | 375 | 384 | i-MoRF | P84092 |
| 20 | P15702 | 20 | 272 | 291 | i-MoRF | P26043 |
| 21 | Q62170 | 18 | 331 | 348 | i-MoRF | P26043 |
| 22 | P20781 | 13 | 420 | 432 | i-MoRF | Q03555 |
| 23 | P05880 | 13 | 662 | 674 | α-MoRF | - |
| 24 | P14416 | 19 | 208 | 226 | i-MoRF | P63096 |
| 25 | Q05586 | 24 | 875 | 898 | α-MoRF | P62161 |
| 26 | P55284 | 16 | 769 | 784 | i-MoRF | Q99NH2 |
| 27 | P46097 | 21 | 40 | 60 | α-MoRF | P10844 |
| 28 | P11836 | 25 | 163 | 187 | complex-MoRF | - |
| 29 | P18508 | 10 | 400 | 409 | i-MoRF | P84092 |
| 30 | O08675 | 13 | 44 | 56 | i-MoRF | P19221 |
| 31 | P21802 | 15 | 764 | 778 | i-MoRF | P21802 |
| 32 | P05106 | 12 | 50 | 61 | i-MoRF | P01903<br>P79483 |
| 33 | Q61391 | 22 | 2 | 23 | β-MoRF | P26043 |

| | | | | | | |
|---|---|---|---|---|---|---|
| 34 | P15379 | 20 | 708 | 727 | β-MoRF | P26043 |
| 35 | P05067 | 10 | 586 | 595 | i-MoRF | P08253 |
| 36 | P01730 | 23 | 428 | 450 | α-MoRF | Q05397 |
| 37 | Q9BY67 | 12 | 400 | 411 | i-MoRF | Q9Y2J2 |
| 38 | Q7SVL4 | 11 | 654 | 664 | i-MoRF | - |
| 39 | Q5S532 | 13 | 649 | 661 | i-MoRF | - |
| 40 | P08138 | 17 | 396 | 412 | α-MoRF | P62158 |
| 41 | Q99523 | 13 | 819 | 831 | i-MoRF | Q9UJY5 |
| 42 | P48050 | 10 | 436 | 445 | i-MoRF | O14907 |
| 43 | P18011 | 22 | 51 | 72 | i-MoRF | P0A2U4 |
| 44 | P03524 | 13 | 512 | 524 | i-MoRF | P29074 |
| 45 | P61014 | 15 | 4 | 18 | i-MoRF | P05132 |
| | | | | | | P05132 |
| 46 | Q9Y696 | 10 | 198 | 207 | i-MoRF | P52293 |
| 47 | P04233 | 19 | 102 | 120 | i-MoRF | P01903 |
| | | | | | | P04229 |
| 48 | Q9NRQ2 | 13 | 271 | 283 | i-MoRF | P52293 |
| 49 | P01130 | 14 | 817 | 832 | i-MoRF | D3ZAR1 |
| 50 | Q9UKV5 | 19 | 622 | 640 | α-MoRF | P55072 |
| 51 | P06974 | 16 | 1 | 16 | α-MoRF | P0AE67 |
| 52 | P02686 | 13 | 221 | 233 | α-MoRF | P01903 |
| | | | | | | Q30154 |
| | | | | | | P0C0I5 |
| 53 | P40338 | 15 | 123 | 137 | i-MoRF | Q03071 |
| 54 | P49418 | 10 | 322 | 331 | α-MoRF | P17427 |
| 55 | P02686 | 15 | 217 | 231 | i-MoRF | P01903 |
| | | | | | | P01911 |
| | | | | | | P01848 |
| | | | | | | P01850 |
| 56 | Q811U3 | 10 | 939 | 948 | complex-MoRF | Q9JIR4 |
| 57 | P0ABB0 | 22 | 1 | 22 | α-MoRF | P0ABA4 |
| 58 | Q9UJY5 | 13 | 376 | 388 | i-MoRF | Q9UJY5 |
| 59 | Q9BY43 | 13 | 210 | 222 | complex-MoRF | Q8WUM4 |
| 60 | Q9H444 | 18 | 207 | 224 | α-MoRF | Q8WUM4 |
| 61 | Q96CF2 | 13 | 221 | 233 | i-MoRF | Q8WUM4 |
| 62 | P03372 | 10 | 298 | 307 | complex-MoRF | Q8WTS6 |
| 63 | P50552 | 16 | 199 | 214 | i-MoRF | P07737 |
| 64 | O60331 | 15 | 639 | 653 | i-MoRF | P62944 |
