# Supplementary figures and images for: MFSPSSMpred: identifying short disorder-to-order binding regions in disordered proteins based on contextual local evolutionary conservation
Source: BMC Bioinformatics. 2013 Oct 4;14:300. doi: 10.1186/1471-2105-14-300 (PMC3853019; doi:10.1186/1471-2105-14-300)

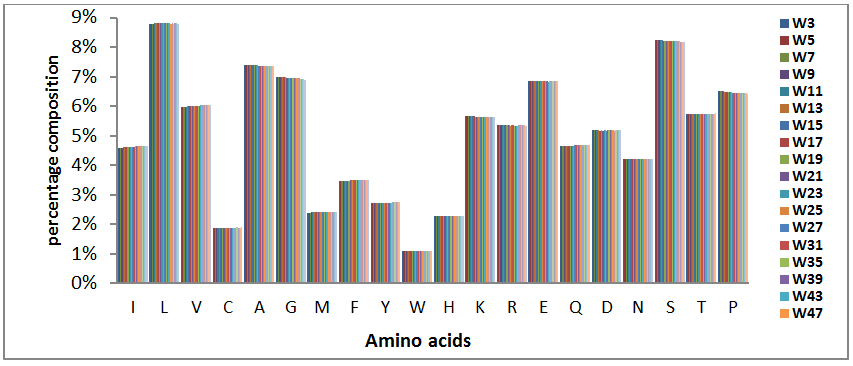

Supplement: Additional file 2: Figure S1 — Composition distribution of general non-MoRF regions with different flanking-window lengths (excluding flanking regions, W indicates the flanking window length). [file 1471-2105-14-300-S2.tif]

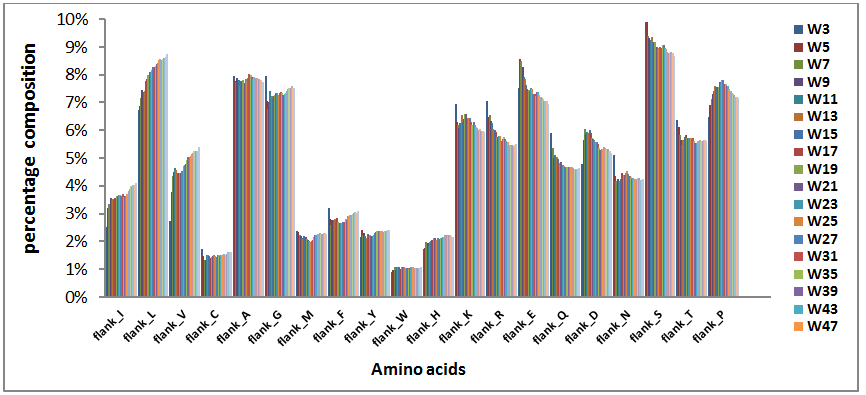

Supplement: Additional file 3: Figure S2 — Composition distribution of the flanking regions. W indicates the flanking window length. [file 1471-2105-14-300-S3.tif]

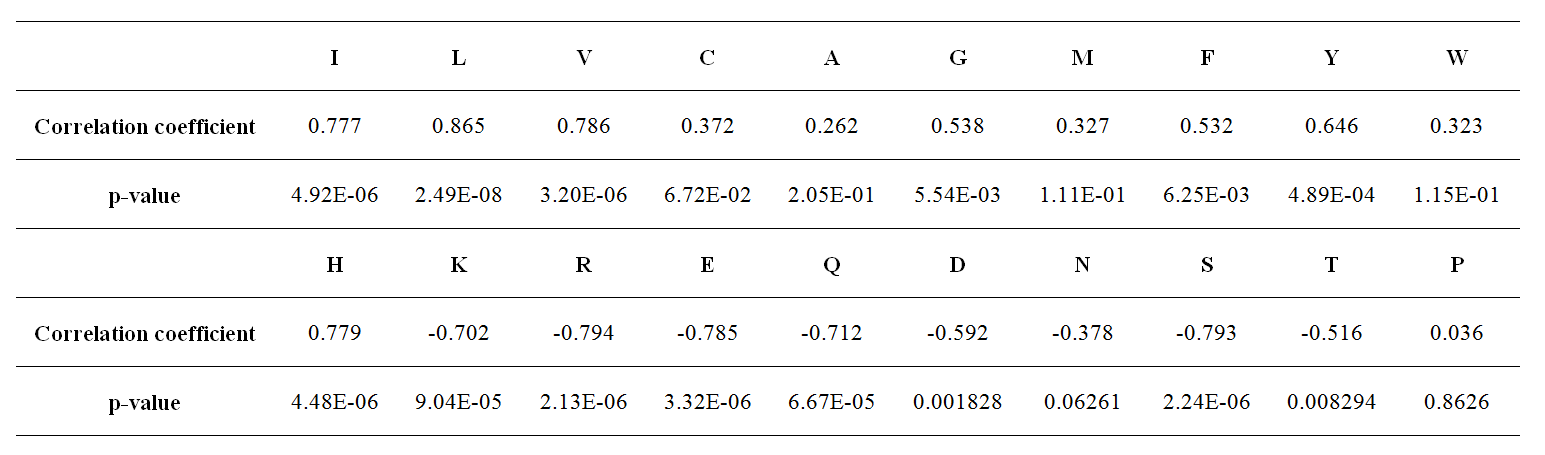

Supplement: Additional file 4: Table S2 — Correlation coefficients between flanking length and composition difference (Flanking regions - general non-MoRF regions) for each amino acid. [file 1471-2105-14-300-S4.tif]

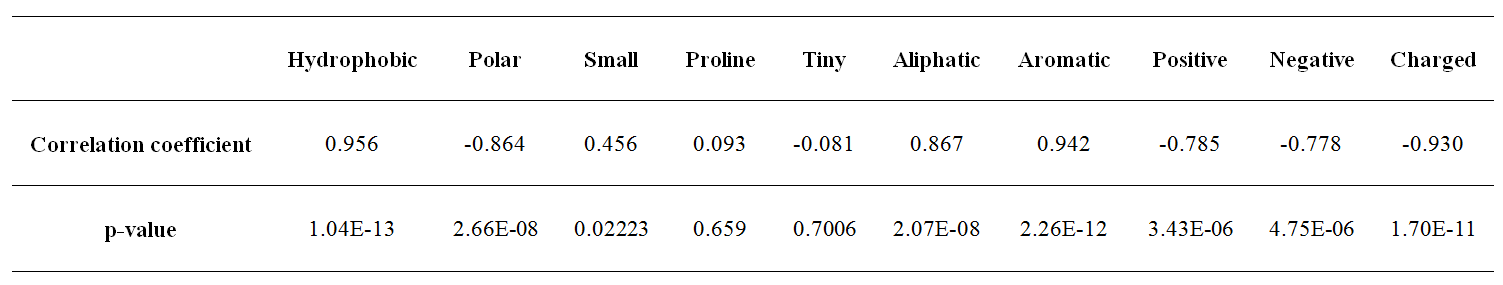

Supplement: Additional file 5: Table S3 — Correlation coefficients between flanking length and physicochemical properties difference (Flanking regions - general non-MoRF regions) for each property. [file 1471-2105-14-300-S5.tif]

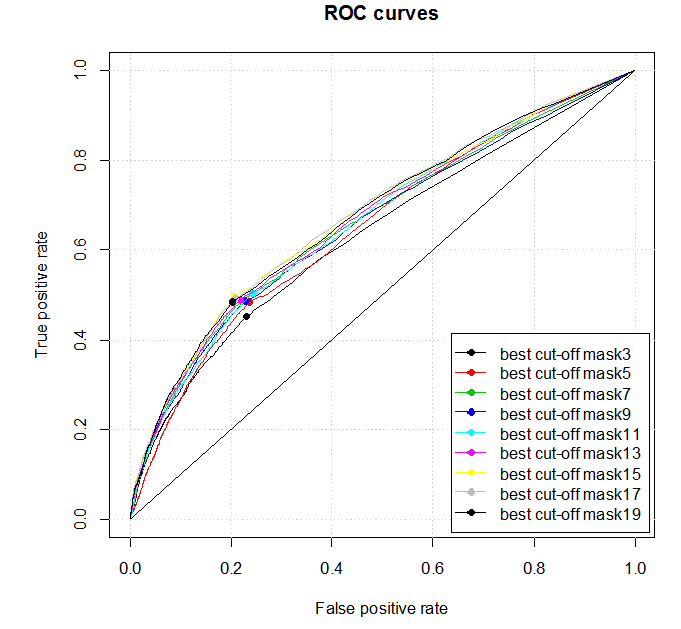

Supplement: Additional file 6: Figure S3 — ROC plots of MFSPSSMpred tested with different masking-window sizes (from 3 to 19). [file 1471-2105-14-300-S6.tif]
